# Factors associated with perceived stress of clinical practice among associate degree nursing students in Taiwan

**DOI:** 10.1186/s12912-021-00602-6

**Published:** 2021-06-07

**Authors:** Chia-Shan Wu, Jiin-Ru Rong, Mei-Zen Huang

**Affiliations:** 1 Department of Nursing, National Tainan Junior College of Nursing, 78, Sec. 2, Min-Tsu Rd, Tainan City, 700 Taiwan R.O.C.; 2 School of Nursing, National Taipei University of Nursing and Health Sciences, 365, Ming-te Rd., Peitou District, Taipei City, 11219 Taiwan R.O.C.

**Keywords:** Nursing students, Clinical practice, Perceived stress, Decision tree

## Abstract

**Background:**

Clinical placements play an important role in helping nursing students to achieve clinical competence, but these placements can be highly challenging and stressful. It has been shown that stress can be either a trigger or aggravating factor for ill-health in general, but studies have seldom differentiated the impact of general health status on perceived stress.

This study examined factors associated with perceived stress of clinical practice among nursing students with a particular focus on the effect of general health status on stress.

**Methods:**

This was a cross-sectional quantitative study conducted among 724 associate nursing degree students in Southern Taiwan.

**Results:**

Health status scores varied from 28 to 139, with an average of 68.40 (SD = 25.75). Health status was reported to be ‘good’ (scores 28–55) in 35.5% of participants, moderate (scores 56–83) in 24.6%, and poor (Scores ≧ 84) in 39.9% of participants. Perceived stress scores ranged from 0 to 95 points with an average score of 36.65 (SD ± 15.95). The classification and regression tree (CART) analysis showed health status as the most important factor linked to perceived stress with a Normalized Importance value of 100%. Those who reported general health status (measured through General Health Questionnaire (GHQ)-28) score of ≤34.5 perceived mild stress and those with a score of > 34.5–< 84.5 perceived moderate stress. A score of 84.5 was found to be the point of transition to perceptions of severe stress. When health status score was greater than 84.5, perceived stress was at a severe or extremely severe level.

**Conclusions:**

Our findings indicated health status as a potential measure to identify students who were most vulnerable to perceived stress. Given the cross-sectional design of this study and the bidirectional relationship between health and stress, more studies are needed to fully establish the predictive link between general health status and vulnerability to stress.

## Introduction

Clinical placements play an important role in helping nursing students to achieve clinical competence, and to develop elements of professional practice in real world scenarios. These placements help students to gain practical experience in taking care of patients and to attain a gradual sense of belonging to the nursing profession, whilst improving their socialization skills and professional role confidence [1, 2]. The first clinical placement is considered to be of paramount importance within nursing students’ educational and professional journey.

While the merits of clinical practice for healthcare students is well established in general, these placements can also be highly challenging and stressful [3–5] and for some nursing students, the perceptions and experiences are markedly inconsistent with the desired outcome [6]. Studies have widely recognised that the transition from being a nursing student to a practitioner working with real patients, involves a range of experiences from just being uncomfortable to undergoing a reality shock along with feelings of self-doubt and inadequacy coupled with discouragement and exhaustion [5, 7–9]. Studies have shown that most students experienced stress due to reasons such as their inability to put clinical theory into practice; fear of making the wrong decisions; unfamiliarity with applying professional knowledge; lack of confidence in implementing clinical skills; poor communication between patients, family members, and professional team members; distrust of patients and their families; pressure during acute medical situations, and poor time management [10–15]. The transition to clinical practice during placement is also indicated to be a great source of anxiety and fear with students experiencing high levels of physical exertion and fatigue due to the pressure from professional interest and expectations [16–19]. Stress during clinical placements may lead to physical and psychological symptoms such as frequent headaches, anxiety, nervousness, poor sleep, lack of attention, cognitive decline, and learning difficulties, that reduce the ability to provide clinical care, and may even affect their willingness to work as a nurse in future [11, 13, 20–26] .

A number of intrinsic personal factors have been linked to experiences of stress among students in general including low self-esteem, low self-confidence, inadequate decision making abilities, poor self-control, tendency to self-blame and poor social behaviours [17, 27–29]. Theoretical models such as the stress vulnerability model has posited that stress in the environment can worsen biological vulnerability to ill health [30]. Studies in the general population have shown that stress can be either a trigger or aggravating factor for ill-health including diseases and pathological conditions [31, 32]. It has also been generally accepted that stress and health could have a bidirectional relationship with higher levels of stress contributing to poor health status or vice versa with poor health status contributing to increased vulnerability to stress. However, studies have rarely been able to differentiate the impact of general health status on vulnerability to experiences of stress. While general health status could be one of the potential factors linked to stress among nursing students during clinical placement, no studies have been conducted to the authors’ knowledge to examine the impact of general health status on the levels of stress.

In Taiwan, clinical placement constitutes a key component of the nursing program with the aim of providing nursing students with the essential practical skills of a registered nurse. In order to be able to register as a nurse in Taiwan, nursing students are required to complete a program of 1016 h of clinical placement in teaching hospitals approved by the Ministry of Examination [33]. After completing the course requirements, nursing students normally undergo a rotational placement through each placement unit for 1 year before graduation. This placement is indicated to have beneficial impact on Taiwanese nursing students’ professional development. For example, a qualitative study exploring nursing students’ perceptions towards the nursing profession based on their experiences of clinical placement in a baccalaureate nursing program indicated that clinical practicum facilitated baccalaureate nursing students’ psychological development and professional knowledge. The experiences gained in real clinical situations during clinical placement helped nursing students to clarify their misconceptions about the nursing profession and to recognize the true nature of the profession while developing cognitive and skill based competencies [34]. Another study on perceived stress and physio-psycho-social status of nursing students during the initial period of clinical practice in Taiwan reported that stress for these students came mainly from the lack of professional knowledge and skills and the most common response to stress was manifestation of social behavioural symptoms [35]. A cross-sectional study of 357 nursing diploma students found that higher perceived stress, female sex, and an introverted personality trait were significant independent factors of physio-psycho-social symptoms during their clinical placement [36]. Studies have, however, been unable to differentiate the impact of general health status on the level of stress experienced during clinical placement.

In order to help nursing students cope with the stress of clinical placement and complete their programs more smoothly, there is a need for a better understanding of perceived stress of clinical practice among nursing students in different cultural settings and to investigate the factors that are linked to stress during placement. The aim of this study was to examine factors associated with perceived stress of clinical practice among nursing students in Taiwan with a particular focus on the effect of general health status on stress.

## Methods

This was a cross-sectional quantitative study conducted among senior students in a five-year associate nursing degree program in a nursing school in Southern Taiwan.

### Participants /sample

The participants comprised of senior students in a five-year associate nursing degree program of a nursing school in southern Taiwan. A convenience sampling approach was used to recruit the participants. The inclusion criteria for participation were that they were either in the fourth or fifth year of the program known as the clinical placement phase, where they spend either full or half year in clinical practice; they did not have any severe physical or mental health problems as diagnosed by a physician; and they were not dealing with a family crisis during the internship period prior to the data collection as reported by a clinical instructor.

According to the “Basic Information of Schools at All Levels” of the Statistics Department of the Ministry of Education [37], the school has a total of 1939 registered students in 2019. The estimated sample size was calculated according to the following formula [38]:

$$ Sample\ size=\frac{\frac{{\mathcal{z}}^2\times p\left(1-p\right)}{e^2}}{1+\left(\frac{{\mathcal{z}}^2\times p\left(1-p\right)}{e^2N}\right)} $$

Assumptions were made as follows: the probability of performing good or bad in students’ clinical practice was *p* = 0.5, the sampling error was 0.03and the confidence level was 0.95. The sampling error was set at 0.03 consistent with the general rule regarding a 3% acceptable margin of error in educational and social research for continuous data [39, 40]. A 3% margin of error would give the researcher the confidence that the true population mean on a seven-point scale is within ±.21 range (.03 × 7) of the mean calculated using the sample. The sample size for the study was calculated to be 689. With an additional 10% nonresponse rate, the final sample size was calculated to be 758.

### Data collection tools

The study instruments included the Perceived Stress Scale for Nursing Students in Clinical Practice [41] and the General Health Questionnaire − 28 [42]. The Perceived Stress Scale, originally developed by Sheu et al. [41] is designed to understand the extent and type of stress as perceived by nursing students in Taiwan. The Scale consisted of 29 items to measure perceived stress, grouped into six domains including: the stress of providing nursing care to patients; stress from teachers and the nursing staff; stress of dealing with paper work and the workload of clinical nursing; stress of dealing with peers and others; stress of demonstrating the mastery of professional knowledge and skills, and stress of the practicum work environment. Each item was graded on a 5-point Likert scale 0 = “never,” 1 = “rare,” 2 = “sometimes,” 3 = “frequent,” and 4 = “always.” The higher the score, the higher the perceived stress. The reliability, content validity and construct validity the test has been demonstrated by Sheu et al. [41].

The Chinese version of the General Health Questionnaire (GHQ) -28 [42, 43] was used to assess the participants’ physical and mental health in the past month. The GHQ-28 is a self-administered instrument, developed based on an exploratory factor analysis (EFA) of the original GHQ-60. Although it was originally developed in English, the questionnaire has been translated into different languages, including Chinese [43], and the stability of the factor structures has been evaluated across different cultures and samples [44–46]. The 28-item GHQ consist of four subscales with 7-items each: somatic symptoms, anxiety and insomnia, social dysfunction and severe depression [42]. The participants indicated symptoms related to their health in general over the past month, using the items on a 5-point Likert scale as follows: never (score = 1), less than usual (score = 2), as usual (score = 3), more than usual (score = 4), much more than usual (score = 5) with higher score showing worse physical and mental health status. The scores were then grouped into three categories to indicate the health status: scores 28–55 (good health), scores 56–83 (moderate health) and scores ≧ 84 (poor health).

### Data collection

After gaining permission from the nursing school as well as ethics approval, the lead author and two research assistants who had received training for data collection personally approached students in their placement clinics. The team explained the study’s purpose and methods to each student individually along with an information sheet. Those who were interested to take part after the initial discussion were given detailed information about the study, and what the participation involved. Each student was also provided with a consent form and a prepaid envelope for returning the consent form. Upon receipt of the signed consent form, the research team visited the placement clinic to hand out the self-administered questionnaires including the Perceived Stress Scale for Nursing Students and the General Health Questionnaire (GHQ) - 28 along with an envelope for return. The students took 1 h on average to complete the questionnaires and returned them in a sealed envelope to the researchers. They completed the questionnaires in a room free from interruptions.

The researchers informed the participants that they could withdraw from the study at any time without affecting their placement or studies. The participants were also informed that they could request their data to be deleted by a certain date even though they had completed the questionnaires, and the data analysis was conducted only after this date. None of the participants requested to delete the data. Students who participated were offered fluorescent markers as a reward for their time.

### Ethical considerations

Ethical approval of the study was obtained from the Ethics Committee of National Cheng Kung University (NCKU HREC-E-105-113-2). The researchers explained the study’s purpose and methods to potential participants. Those who were interested to take part after the initial discussion were given detailed information about the study in a cover letter, and what the participation involved. The researchers assured the participants that the information collected will be kept confidential and anonymous. Potential participants were also made aware of their right to refuse to participate in or to withdraw at any stage during the course of the study.

### Statistical analysis

The IBM Statistical Package for Social Sciences (SPSS) for Windows, version 23.0 (Armonk, NY: IBM Corp.) was used to perform the statistical analysis. Data were summarized as mean and standard deviation for continuous variables. The variables were normally distributed. The Pearson’s correlation coefficient was used to analyse associations, with *p* values <.05 regarded as significant. A multi-factor analysis of variance (ANOVA) procedure was performed for group comparisons.

A classification and regression tree (CART) analysis was conducted to develop a model to understand the effect of factors such as general health status, internship category, grade of intern, gender and sleeping hours on levels of perceived stress. CART is an explanatory technique that has been widely used to reveal data structure, identify important characteristics, and develop decision trees [47]. The CART model is composed of three main steps: (1) CART initialization: generating a decision tree based on training data set; (2) CART pruning and optimization: the regression tree is pruned according to constraints, such as the maximum depth of the tree, the minimum sample size of the leaf node and the node’s minimum impurity as the model has best generalization through the combination of different parameters that generated different CART models - the maximum depth of the tree (max depth), the minimum sample number of leaf nodes (min_samples_leaf), the minimum impurity of the nodes (min_impurity_split) (3) CART prediction: put the test set into the trained model and creating prediction [48].

The CART analysis was conducted in three steps: 1. A classification model was constructed from the entire sample to create the largest tree structure with the minimum sample of the parent code as 100 and the minimum sample of the child node as 50; 2. The correct rate of the classification model was evaluated by using K- fold cross-validation with the value set at 10; 3. The maximum difference in risk was used in post-pruning and the value was set to 0 in order to generate the tree structure with minimum risk value. A decision node was taken as the point for making the decision, with the branches drowning from a node representing the possible alternatives or courses of action available at that point. The set of alternatives were considered mutually exclusive and collectively exhaustive [49–51].

## Results

### Demographic characteristics

A total of 790 questionnaires were distributed to eligible respondents in the associate nursing degree program and 724 completed questionnaires were returned with an overall response rate of 91.6%. The demographic characteristics of the participants are presented in Table 1. The majority of participants were females (96.7%). Their average age was 19.13 (SD ±0.42) years and a great majority (79.4%) were in the final year of the degree programme. While more than half of the students (57.6%) reported their sleeping hours as 6–8 h, 40.9% of the students slept for less than 5 h per day. The practice areas included Medical Surgical Nursing (13.54%), Obstetrics Nursing (16.16%), Paediatrics Nursing (18.23%), Psychiatric Nursing (19.47%), Community Nursing (19.06%) and Long-Term Care (13.54%). Majority (79.42%) of the students had been in internship for 6 months and 20.58% had been in internship for 2–4 months at the time of data collection.

**Table 1:** Demographic data (*N* = 724)

Variable	Numbers	%
Gender		
Male	24	3.3
Female	700	96.7
Practice grade		
Fourth grade	149	20.6
Fifth grade	575	79.4
Sleep duration (hours)		
9–10	11	1.5
6–8	417	57.6
4–5	288	39.8
1–3	8	1.10
Practice area		
Medical Surgical nursing Practicum	98	13.54
Obstetrics nursing Practicum	117	16.16
Pediatric Nursing Practicum	132	18.23
Psychiatric nursing Practicum	141	19.47
Community Nursing Practicum	138	19.06
Long-Term care Practicum	98	13.54
Duration of clinical practice (months)		
2–4	149	20.58
6	575	79.42

### Nursing students’ health status

The health status scores among the participants varied from 28 to 139, with higher score indicating worse health status. The average score was 68.40 (SD = 25.75). The worst health status was reported on domains of anxiety and insomnia (Mean: 24.97; SD: ±4.35) and social dysfunction (Mean: 24.31; SD: ±3.26). (Table 2). Based on the overall scores, the health status was deemed to be ‘good’ (scores 28–55) in 35.5% (*n* = 257) of the participants, moderate (scores 56–83) in 24.6% (*n* = 178) participants, and poor (Scores ≧ 84) in 39.9% (*n* = 289) participants. The most common areas reported indicating poor health status were: “Been satisfied with the way you have carried out your tasks” (Mean 2.89; SD ± 1.14) “Been feeling capable of making decisions about things,” (Mean 2.86; SD ± 1.14) “Been managing to keep yourself busy and occupied,” (Mean 2.41; SD ± 1.09) “Been feeling everything is getting on top of you.” (Mean2.77; SD ± 1.21) (Table 3).

**Table 2:** Students’ health status scores on individual domains

Domain	Good (Scores 28–55) (35.5%, *n* = 257)		Moderate (Scores 56–83) (24.6%, *n* = 178)		Poor (Scores ≧ 84) (39.9%, *n* = 289)		Overall (*n* = 724)	
	M ± SD	Range	M ± SD	Range	M ± SD	Range	M ± SD	Range
Somatic symptoms	10.11 ± 3.36	7–22	18.33 ± 4.32	7–34	23.57 ± 3.86	16–67	17.45 ± 6.74	7–35
Anxiety and insomnia	9.69 ± 3.42	7–23	19.8 ± 4.42	7–30	24.97 ± 4.35	19–68	18.24 ± 7.60	7–35
Social dysfunction	11.05 ± 4.13	7–23	19.76 ± 4.23	9–45	24.31 ± 3.26	15–35	18.47 ± 6.87	7–35
Severe depression	7.77 ± 2.24	7–21	12.44 ± 5.16	7–25	20.93 ± 4.35	8–35	14.17 ± 7.03	7–35

**Table 3:** Students’ health status scores on individual items

Health status factor/item	M	SD	Range	Item ranking	Factor ranking
**I. Somatic symptoms**	17.45	6.74	7–35		3
1. Been feeling perfectly well and in good health?	2.71	1.09	1–5	7	
2. Been feeling in need of a good tonic?	2.35	1.19	1–5	19	
3. Been feeling run down and out of sorts?	2.49	1.16	1–5	12	
4. Been feeling that you are ill?	2.71	1.15	1–5	6	
5. Been getting any pains in your head?	2.46	1.17	1–5	16	
6. Been getting a feeling of tightness or pressure in your head?	2.60	1.22	1–5	11	
7. Been having hot or cold spells?	2.14	1.08	1–5	22	
**II. Anxiety and insomnia**	18.24	7.60	7–35		2
8.Been losing much sleep over worry?	2.48	1.21	1–5	14	
9.Been having difficulty in staying asleep once you fall asleep?	2.48	1.22	1–5	13	
10.Been feeling constantly under strain?	2.74	1.24	1–5	5	
11.Been getting edgy or bad tempered?	2.65	1.21	1–5	10	
12.Been getting scared or panicky for no reason?	2.41	1.19	1–5	17	
13.Been feeling everything is getting on top of you?	2.77	1.21	1–5	4	
14.Been feeling nervous and strung-out all the time?	2.69	1.25	1–5	8	
**III. Social dysfunction**	18.47	6.87			1
15.Been managing to keep yourself busy and occupied?	2.85	1.19	1–5	3	
16.Been taking longer over the things you do?	2.69	1.19	1–5	9	
17.Been satisfied with the way you have carried out your tasks?	2.89	1.14	1–5	1	
18. Been feeling capable of making decisions about things?	2.86	1.14	1–5	2	
19.Been able to enjoy your normal day-to-day activities?	2.48	1.10	1–5	15	
20.Been managing to keep yourself busy and occupied?	2.41	1.09	1–5	18	
21.Been taking longer over the things you do?	2.29	1.12	1–5	20	
**IV. Severe depression**	14.17	7.03	1–5		4
22.Been thinking of yourself as a worthless person?	2.11	1.11	1–5	23	
23.Been feeling that life is entirely hopeless?	2.10	1.12	1–5	24	
24.Been feeling that life is not worth living?	2.06	1.11	1–5	25	
25.Been thinking of the possibility that you may do away with yourself?	1.94	1.06	1–5	26	
26.Been feeling at times that you could not do anything because your nerves were too bad?	2.24	1.18	1–5	21	
27.Been finding yourself wishing you were dead and away from it all?	1.87	1.05	1–5	27	
28.Been finding that the idea of taking your own life keeps coming into your mind?	1.85	1.03	1–5	28	
Overall	68.4	25.75	28–139	–	–

### The perceived stress of clinical practice among nursing students

The perceived stress of clinical practice scores ranged from 0 to 95 points with an average score of 36.65 (SD ± 15.95). Stress was mild (average single-item score, 1 point) in 26.7% (*n* = 193) of students, moderate (average single-item score, 1.5 points) in 64.5% (*n* = 467) of students, and moderate to severe (average single-item score > 1.5 points) in 8.8% (*n* = 64) of students (Table 4). On average, highest scores were reported on domains of stress of ‘taking care of patients’ (Mean:12.83; SD: ±5.14) and the ‘stress from assignments and workload’ (Mean:7.62; SD: ±3.91) ‘teachers and nursing personnel’(Mean:5.90; SD: ±3.47) (Table 4).

**Table 4:** Areas of stress perceived by students – findings from ANOVA

	Mild (Scores 0–29, *n* = 193)		Moderate (Scores 30–43, *n* = 467)		Moderate to Severe (Scores ≧ 44, *n* = 64)		Overall (*n* = 724)		*p*
	M ± SD	Range	M ± SD	Range	M ± SD	Range	M ± SD	Range	
Taking care of patients	7.513 ± 3.823	0–17	14.015 ± 3.504	5–23	20.828 ± 4.907	13–48	12.834 ± 5.144	0–29	< .001
Teachers and nursing personnel	2.383 ± 1.837	0–8	6.539 ± 2.346	0–17	11.891 ± 3.030	5–19	5.900 ± 3.468	0–19	< .001
Assignments and workload	3.469 ± 2.367	0–11	8.441 ± 2.635	3–19	14.109 ± 2.437	9–20	7.618 ± 3.908	0–20	< .001
Peers and daily life	1.166 ± 1.292	0–6	3.507 ± 1.839	0–9	6.719 ± 2.746	0–14	3.165 ± 2.356	0–14	< .001
Lack of professional knowledge and skills	2.187 ± 1.523	0–6	4.703 ± 1.560	0–15	7.000 ± 1.380	3–10	4.220 ± 2.033	0–10	< .001
The clinical environment	.876 ± 1.139	0–4	3.244 ± 1.453	0–8	6.234 ± 1.892	2–10	2.877 ± 2.042	0–10	< .001

Situations that were perceived as most stressful for students were in the area of providing care to patients especially the ‘lack of experience and ability in providing nursing care and in making judgments’, (Mean1.94; SD ± 0.89) ‘inability to reach one’s expectations dealing with challenges arising from the gap between clinical performance and self-expectation’ (Mean1.87; SD ± 0.92,) ‘inability to provide appropriate responses to doctors’, teachers’, and patients’ question’ (Mean1.77; SD ± 0.81,). Worry about grades was also an area of relatively more perceived stress for students (Mean1.76; SD ± 1.08). The domains that were perceived to cause the least stress were the stress from the environment and stress from peers and daily life (Table 5).

**Table 5:** Sources of stress perceived by nursing students

Stress factor/item	M	SD	Range	Item ranking	Factor ranking
**I. Stress from taking care of patients**					1
Lack of experience and ability in providing nursing care and in making judgments (Q2)	1.94	0.89	0–4	1	
Lack of knowledge about how to help patients with physio-psycho-social problems(Q3)	1.74	0.89	0–4	5	
Do not know how to communicate with patients(Q4)	1.41	0.86	0–4	13	
Worry about not being trusted or accepted by patients or patients’ family(Q8)	1.47	0.91	0–4	11	
Experience difficulties in changing from the role of a student to that of a nurse(Q9)	1.12	0.85	0–4	17	
Inability to provide appropriate responses to doctors’, teachers’, and patients’ question(Q10)	1.77	0.81	0–4	3	
Inability to reach one’s expectations dealing with challenges arising from the gap between clinical performance and self-expectation (Q11)	1.87	0.92	0–4	2	
Unable to provide patients with good nursing care(Q12)	1.50	0.76	0–4	9	
**II. Stress from teachers and nursing staff**					4
Experience discrepancy between theory and practice (Q1)	1.62	0.89	0–4	7	
Feel that teachers do not give fair evaluation on students (Q14)	0.73	0.78	0–4	26	
Medical personnel lack empathy and are not willing to help(Q17)	0.72	0.73	0–4	27	
Do not know how to discuss patients’ illness with teachers, and medical and nursing personnel(Q18)	1.12	0.85	0–4	18	
Feel stressed that teacher’s instruction is different from one’s expectations(Q20)	1.04	0.81	0–4	19	
Lack of care and guidance from teachers(Q25)	0.68	0.73	0–4	28	
**III. Stress from assignments and workload**					2
Worry about bad grades(Q13)	1.76	1.08	0–4	4	
Feel that the requirements of clinical practice exceed one’s physical and emotional endurance (Q15)	1.34	0.88	0–4	14	
Experience pressure from the nature and quality of clinical practice (Q16)	1.70	1.09	0–4	6	
Feel that dull and inflexible clinical practice affects one’s family and social life (Q19)	1.49	1.05	0–4	10	
Feel that one’s performance does not meet teachers’ expectations (Q22)	1.31	0.88	0–4	15	
V. Stress from peers and daily life					6
Feel that clinical practice affects one’s involvement in extracurricular activities (Q5)	0.84	1.00	0–4	24	
Feel pressure from teachers who evaluate students’ performance by comparison (Q21)	0.76	0.79	0–4	25	
Cannot get along with other peers in the group(Q23)	0.67	0.73	0–4	29	
Experience competition from peers in school and clinical practice(Q24)	0.89	0.81	0–4	23	
**IV. Stress from lack of professional knowledge and skills**					3
Unfamiliar with patients’ diagnoses and treatments(Q6)	1.42	0.76	0–4	12	
Unfamiliar with medical history and terms(Q7)	1.51	0.82	0–4	8	
Unfamiliar with professional nursing skills(Q26)	1.29	0.83	0–4	16	
**V. Stress from the environment**					5
Feel stressed from the rapid change in patient’s condition(Q27)	0.93	0.72	0–4	22	
Unfamiliar with the ward facilities(Q28)	0.95	0.79	0–4	21	
Feel stressed in the hospital environment where clinical practice takes place(Q29)	1.00	0.90	0–4	20	
Overall	36.65	15.95	0–95	–	–

### Correlation between nursing students’ health status and the level of stress

In the unadjusted analysis of liner correlation between health status and perceived stress, health status was highly positively correlated with the level of stress (*r* = .665, *p* < .01); the more severe the health problems were, the greater the perceived stress was (Table 6).

**Table 6:** Adjusted analysis of the correlation of health status with internship stress (*N* = 724)

	Average	Standard deviation	*r*
Health status	68.40	25.75	.665*
Internship stress	36.65	15.95	

* *p* < .01

### Factors associated with perceived stress of clinical practice: findings from the CART analysis

**Fig. 1:**
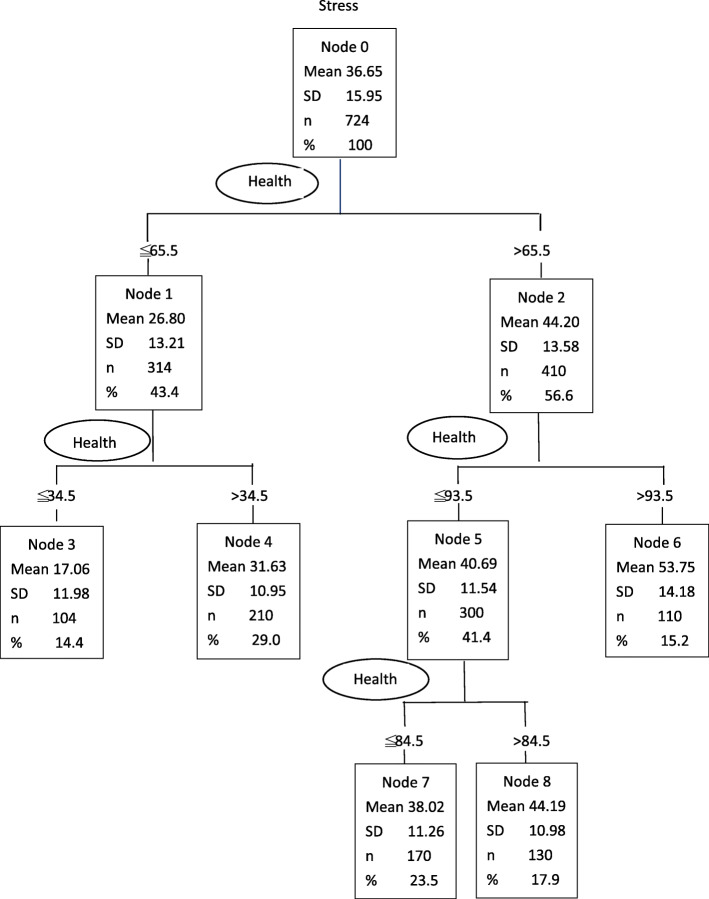
CART analysis: Health status and the level of perceived stress

**Fig. 2:**
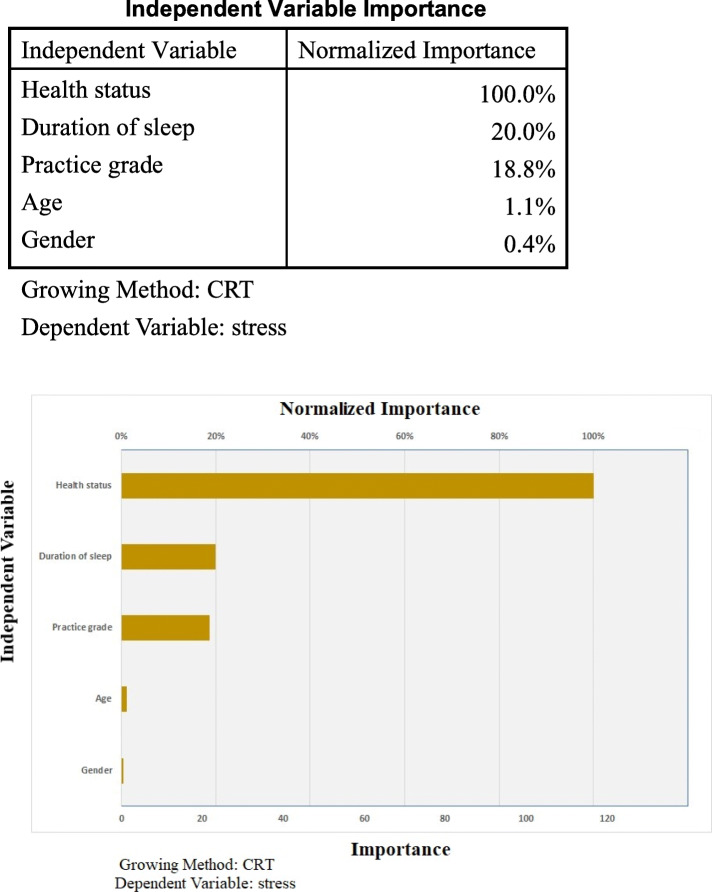
CART analysis: Factors associated with perceived stress of clinical practice

The CART analysis produced a tree model with three branches and five terminal nodes with values for factors associated with experiences of perceived stress. The results of the CART analysis are shown in Fig. 1. The most appropriate tree for examining factors linked to perceived stress of clinical practice consisted of eight nodes and five terminal nodes. On the basis of the minimum Gini improvement measure of perceived stress, we found that general health status was the most highly associated factor in the first layer. The general health status score, predicted score for perceived stress, and population size respectively were < 34.5, 17.06, and 104 at node 3; < 34.5, 31.63, 210 at node 4; ≦84.5, 38.02, and 170 at node 7; > 84.5, 44.19, and 130 at node 8; and > 93.5, 53.75, and 110 at node 6. The analysis showed health status as the most important factor linked to perceived stress with a Normalized Importance value of 100% (Fig. 2). Those who have had a GHQ-28 score of ≤34.5 perceived mild stress and those who have had a GHQ-28 score of > 34.5–< 84.5perceived moderate stress. The GHQ score of 84.5 was found to be the point of transition to severe stress. When health status score was greater than 84.5, perceived stress was at a severe or extremely severe level, indicating that health status was the most predictive factor (Fig. 1).

## Discussion

This study aimed to examine factors associated with perceived stress during clinical practice among nursing students, students in Taiwan with a particular focus on the effect of general health status on stress. To the authors’ knowledge, this is the first study that has examined the impact of general health status on the levels of stress experienced among nursing students during their clinical practice.

More than one third of the students in our study reported poor health status, with the domains of anxiety and insomnia and social dysfunction as the poorest areas of health with dissatisfaction expressed in aspects such as the way tasks were carried out, inability to make decisions, managing to keep themselves busy and occupied, and feeling that everything is getting on top of them. General health is one of the most important factors of student success and academic achievement and nursing students’ general health status during clinical placement will have a great impact on students’ clinical performance, and overall achievement of the learning outcomes. It has been recognised that the health and well-being of student nurses is of considerable importance given the demands posed by the course and the profession, as well as the implications on providing good quality care [52].

Majority of the students in our study experienced moderate to severe levels of stress, with the highest stress experienced in the domains of patient care, assignments and workload and teachers and nursing personnel. Studies have suggested that clinical placements are substantial sources of stress for nursing students [4, 10–15, 27]. Previous researchers have reported specific stressors such as the gap between theory and practice, feelings of lack of preparation for practice or to cope with knowledge and skill demands, fear of making mistakes, fear of death and dying, problematic interpersonal relationships with clinical teachers and nursing staff, lack of familiarity with the clinical environment, and conflicts between professional beliefs and the reality in hospital practice [27, 53, 54]. Previous studies from Taiwan have indicated the link between low interest in clinical practicum placement and increased stress levels in nursing students [55]. The students in our study perceived factors such as lack of experience and ability in providing nursing care and in making judgments, expectations dealing with challenges arising from the gap between clinical performance and self-expectation, lack of knowledge about how to help patients with physio-psycho-social problems and worry about grades as the predominant sources of stress. Although some of these stressors were unavoidable due to students’ limited professional knowledge and clinical skills, the findings indicated the need for teachers and placement supervisors to provide better guidance to students and to prepare them adequately for clinical practice in order to minimise the stress experienced by students.

A number of studies have reported the clinical environment as a key source of stress for students [27, 28, 36, 56, 57]. This was not the case for the participants in our study as we found that the domains that were perceived to cause the least stress for our participants were the stress from the environment and stress from peers and daily life. Similar findings have been reported among nursing students in Taiwan who had completed their initial clinical practice at the largest nursing school [35].

We found that the overall health status was the most significant factor associated with the level of stress experienced by the students; the more severe the health problems were, the greater the perceived stress was. As previous studies have not focused on the effect of health status on perceived stress during clinical practice in Taiwanese or other similar settings, it is difficult to directly compare the findings with those from previous studies. The link between current health status scores and vulnerability to stress that we found in our study is an important finding as this measure enables to identify students who are most vulnerable to stress.

A major limitation of this study was the use of convenience sampling and the lack of diversity as participants were recruited from the associate degree nursing program in one nursing school and this could limit the wider applicability of the findings. In addition, the cross-sectional nature study would have limited the casual inferences between health status and perceived stress. Given the cross-sectional nature of the study design and the potential bidirectional relationship between health status and stress, the observed predictive value of health status on stress need to be treated with caution as it is difficult to infer whether poor health status caused vulnerability to stress or higher levels of stress contributed to poor general health status. The use of self-reported data might have also affected the validity of the findings.

## Conclusion

Overall, the findings from the current study provided key insights into perceived stress of clinical practice among final year nursing students in Taiwan and demonstrated the link between health status and vulnerability to stress. The study also added to the existing body of evidence about the potential use of GHQ-28 to assess nursing students’ vulnerability to stress during clinical placement and how general health status could be used as a measure to identify students who are most vulnerable. However, given the nature of cross-sectional design of this study and the bidirectional relationship between health and stress, more studies are needed to establish the predictive link between general health status and vulnerability to perceived stress.

The findings have important implications for nursing educators as well as placement supervisors in identifying and supporting students who are vulnerable to experience stress during clinical practice. As some of the stressors are inevitable, nursing students should be provided with appropriate educational as well as other evidence based interventions towards boosting their general health and well-being during clinical placement thereby enhancing their ability to cope with the stress of clinical practice. As the health of an individual is dependent upon a range of internal and external factors, it is of considerable importance to examine and continuously monitor factors that contribute to poor health at an individual level throughout their studies as well as during the practicum.

The psycho-social support from peers and family members is another aspect to consider and build on when planning psychosocial intervention programmes. Future studies should examine the longitudinal link between health status and perceived stress using experimental designs as well as explore coping behaviours at different stages of clinical placement and the feasibility of introducing appropriate longitudinal interventions. The perspectives of educators and placement supervisors about potential sources of stress are also worth exploration.

## Data Availability

The datasets used and/or analysed during the current study are available from the corresponding author on reasonable request.

## Abbreviations

CART: Classification and regression tree
GHQ: General Health Questionnaire
EFA: Exploratory factor analysis
